# Advanced Coatings with Antioxidant and Antibacterial Activity for Kumquat Preservation

**DOI:** 10.3390/foods11152363

**Published:** 2022-08-07

**Authors:** Mile Li, Mengyi Wang, Shuhui Hu, Jing Sun, Mingqiang Zhu, Yongsheng Ni, Jianlong Wang

**Affiliations:** 1 College of Food Science and Engineering, Northwest A&F University, Yangling 712100, China; 2 Qinghai Provincial Key Laboratory of Qinghai-Tibet Plateau Biological Resources, Northwest Institute of Plateau Biology, Chinese Academy of Sciences, Xining 810008, China; 3 College of Mechanical and Electronic Engineering, Northwest A&F University, Yangling 712100, China; 4 School of Food and Biological Engineering, Hefei University of Technology, Hefei 230601, China

**Keywords:** coatings, fruit preservation, konjac glucomannan, curcumin, phloretin

## Abstract

An active coating is one of the best ways to maintain the good quality and sensory properties of fruits. A new active coating was prepared by incorporating curcumin and phloretin into the konjac glucomannan matrix (KGM-Cur-Phl). The fourier infrared spectroscopy, rheology and differential scanning calorimetry confirmed the successful fabrication of this coating. This coating showed excellent antioxidant activity revealed by the 95.03% of ABTS radicals scavenging ratio and 99.67% of DPPH radicals scavenging ratio. The result of bacteria growth curves showed that it could effectively inhibit the growth of *Staphylococcus aureus*, *Escherichia coli*, *Listeria monocytogenes* and *Salmonella typhimurium*. The results of firmness, titratable acid and pH showed that it effectively prolonged the shelf life of kumquat. A novel konjac glucomannan-based active coating was provided.

## 1. Introduction

In recent years, with the improvement of living standards, consumers now pay more attention to food quality. Consumers’ concerns about food corruption in the process of food processing, transportation and storage have made people aware that food packaging not only acts as a barrier, but it also has a protective function [1]. Therefore, as a new type of packaging, functional packaging coatings have attracted extensive attention. Functional packaging coatings refers to the use of food packaging materials with special protective properties for food preservation. These food packaging materials show special protective properties including gas absorption, oxidation resistance and antibacterial properties [2]. In addition, due to the concern about environmental issues, environmentally friendly food packaging is very popular. Therefore, when selecting functional packaging materials, consumers not only consider whether the materials can ensure the quality and extend the shelf life of a product, but also consider whether they are green and safe. In recent years, natural functional active substances have been added to biodegradable polymers to obtain active coatings, introducing new ideas to the research of scientists and the food industry [3]. Biodegradable polymers, especially polysaccharides, are recognized as green food packaging materials around the world [4]. At the same time, adding natural active substances with different functions to basic materials is the development trend of active packaging. Edible films based on polysaccharides are considered to be safe and environmentally friendly food packaging materials [5]. Therefore, new active coatings developed by combining polysaccharide with natural active ingredients are worthy of in-depth study.

Konjac glucomannan (KGM) is a non-ionic hydrophilic polysaccharide extracted from Konjac tubers. Natural plant extracts have good biocompatibility, safety, non-toxicity, editability, biodegradability and coating properties. In addition, they have the characteristics of rich resources, easy access, recyclability and good physical and chemical inertia, which shows that KGM can be used as a good skeleton material for the preparation of packaging [6]. Therefore, driven by consumer preferences and their own characteristics, food coatings based on KGM have broad development prospects. However, pure KGM has poor active capacity, which hinders its rapid development. Kumquat is a traditional fruit with high nutritional value and a delicious taste. Kumquat is widely planted in the Qinling Mountains and south of the Yangtze River. Because of its golden smooth peel, crisp and juicy pulp and unique aroma, it has become a popular fruit. In addition, its high medicinal value and strong antioxidant activity improve its market value. If kumquat is not properly preserved in the process of production and sales, it will cause huge economic losses. Therefore, kumquat was chosen as the representative object to explore the effects of this active coating on the freshness of fruit. In addition, as Kumquat is a fruit that can be consumed raw without peeling, its preservation method must be efficient, appropriate, safe and non-toxic.

Curcumin (Cur) is a natural polyphenol compound found in turmeric [7]. Because of its excellent antibacterial, antioxidant effects and edibility, it is widely used in the field of food [8]. Unfortunately, the low water solubility of curcumin hinders its application in food [9]. Phloretin (Phl) is a natural dihydrochalcone found in fruits and vegetables [10]. It has a variety of pharmacological effects, the most prominent of which is antibacterial and antioxidant activity [11]. However, the application of Phl is limited due to it poor solubility. As a non-ionic hydrophilic polysaccharide, KGM has become an excellent carrier matrix in the food industry [12].

**Figure 1:**
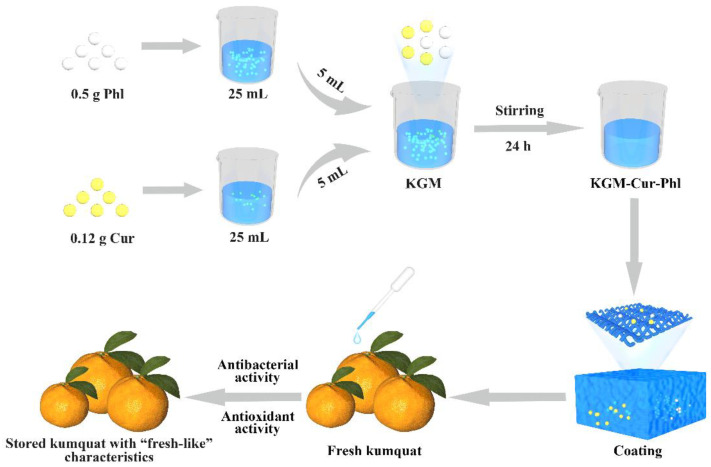
Preparation process of konjac glucomannan coatings and its application in kumquat preservation.

Here, an active coating composed of KGM, Cur and Phl was successfully fabricated. On one hand, KGM can weaken the insoluble shortcomings of Cur and Phl. On the other hand, Cur and Phl can improve the antibacterial and antioxidant activity of KGM. The FTIR, DSC, antioxidant activity, antibacterial activity and the effect of coatings on the freshness of fruit were studied (Figure 1).

## 2. Materials and Methods

### 2.1. Materials

Konjac glucomannan (KGM, Mw = 1.06 × 10^6^ Da, purity ≥ 95%) was obtained from Shaotong Sanai Konjac Development Co., Ltd., (Yunnan, China). Curcumin and phloretin were purchased from Aladdin (Shanghai, China). Kumquats (produced in Qinling Mountains and south of the Yangtze River) were bought from the Hao You Duo supermarket (Yangling, China). The experimental water was deionized water.

### 2.2. Preparation of Konjac Glucomannan Coatings

First, 120 mg of curcumin and 500 mg of phloretin were dispersed into 25 mL of water, respectively, to form a turbid liquid. Then, 0.25 g of KGM was dissolved into 25 mL deionized water by stirring for 5 h to form KGM sol. Finally, 5 mL of curcumin and 5 mL of phloretin turbid liquid were added dropwise to 25 mL of KGM sol. This solution was homogenized (5000 rpm, 5 min) and further stirred continuously for 24 h to form KGM-curcumin-phloretin coatings (denoted as KGM-Cur-Phl). The pure KGM, KGM-curcumin coatings and KGM-phloretin coatings were also prepared (denoted as KGM, KGM-Cur and KGM-Phl, respectively). The kumquat without coating was the control sample.

### 2.3. Characterization of Konjac Glucomannan Coatings

The composition of the coating was investigated by fourier transform infrared spectrometer (FTIR). The measured range was from 4000 cm^−1^ to 400 cm^−1^ at a resolution of 4 cm^−1^ (Vertex 70, Bruker, Germany). The rheological properties of the coating were evaluated using a rheometer (DHR-1, TA Instruments, New Castle, DE, USA) at 25 °C according to the previously reported method [13]. Differential scanning calorimetry (DSC, Q2000, Waters Technology Co., Ltd., Worcester, MA, USA), a thermal analysis method, was used to measure the thermal stability of coatings from 25 °C to 300 °C at a heating rate of 5 °C/min under nitrogen flow (25 mL/min).

### 2.4. Antioxidant Activity of Konjac Glucomannan Coatings

According to the method described by previous studies [14], the antioxidant activity of active coatings was evaluated. The sample (200 μL) was blended with DPPH solution (0.2 mM), which was placed at 20 °C for 30 min in the dark. Then, the absorbance of the above solution was measured at 517 nm and the scavenging activity was calculated by the following equation:

(1) $$1-\frac{A_{1}-A_{2}}{A_{0}}\times 100$$

where *A*_0_ is the absorbance of the DPPH, *A*_1_ is the absorbance of the mixture, *A*_2_ is the absorbance of the methanolic sample.

For ABTS scavenging activity, it used the method from previous studies [15]. The mixture of ABTS (7 mM) and potassium persulfate (2.45 mM) was placed in the dark for 12–16 h and diluted to the absorbance of 0.7 at 734 nm to obtain the ABTS solution. Then, the sample (200 μL) was added to ABTS solution (4 mL), which was put in the dark for 20 min. The absorbance was measured, and the corresponding free radical scavenging activity was calculated according to following equation:

(2) $$\mathrm{Free}\;\mathrm{radical}\;\mathrm{scavenging}\;\mathrm{activity}\;(\%)=1-\frac{A_{sample}}{A_{control}}\times 100$$

where the *A_sample_* is the mixture of the ABTS solution and the sample, the *A_control_* is the initial ABTS solution.

### 2.5. Antibacterial Activity of Konjac Glucomannan Coatings

The growth curve of bacteria was determined to reveal the antibacterial activity of konjac glucomannan coatings. *Escherichia coli* (*E. coli*, ATCC 25922), *Listeria monocytogenes* (*L. monocytogenes*, CICC 21635), *Salmonella typhimurium* (*S. typhimurium*, ATCC50115), and *Staphylococcus aureus* (*S. aureus*, ATCC 29213) were employed to measure the antibacterial activity of coatings. Briefly, referring to the modified protocol [16], the slow-growing bacterial cells were cleaned with phosphate-buffered solution (PBS, 0.01 M, pH 7.4), then it was resuspended and diluted to 1 × 10^8^ CFU/mL in fresh Luria-Bertani (LB) broth. Afterwards, the coating solutions was mixed with bacteria fluid by the ratio 4:1 (V/V) and cultured at 37 °C. Finally, the growth curve was obtained by measuring absorbance under 600 nm. The control was bacteria fluid without treatment.

### 2.6. The Preservation Experiment on Kumquats

The preservation experiment on kumquats was carried out with reference to the previous method [17]. After washing with sterilized water, kumquat was divided into the following five subgroups: KGM, KGM-Cur, KGM-Phl, KGM-Cur-Phl and control group. The kumquat was covered by the corresponding coatings except for the control group and stored at 20 ± 1 °C and 75 ± 1% of the relative humidity. Then, the representative pictures were collected. The firmness of kumquat was obtained by TA-XTPlus texture analyzer (Stable Micro Systems, Landon, UK). After wiping the coatings on the surface of the kumquat, the corresponding juice was taken. The pH value and DPPH radicals scavenging ratio of kumquats juice also were measured. In addition, the titratable acid was measured according to the following steps:

Referring to the previous method [18], the coated fruits (45 ± 2.5 g) were mashed, homogenized and diluted with 5 mL of deionized water. Then,1 mL of supernatant, 18.8 mL of distilled water and 0.2 mL of phenolphthalein reagent (1%) were added into the triangular flask. Finally, the solution was titrated with NaOH (0.05 M) to reddish and did not fade for 30 s. The titratable acid was acquired according to the following equation:

(3) $$\mathrm{Titratable}\;\mathrm{acid}\;\mathrm{content}\;(\%)=\frac{c\times \left(V_{1}-V_{2}\right)\times 0.067\times F}{m}\times 1000$$

where *V*_1_ is the volume of NaOH used in blank experiment, *V*_2_ is the volume of NaOH used in titration experiment, *F* is the dilution multiple of sample, m is the weight of the titration sample.

### 2.7. Statistical Analysis

All results were statistically evaluated by an SPSS software (SPSS 23.0 for windows, SPSS Inc., Chicago, IL, USA).

## 3. Results

### 3.1. Characterization of Konjac Glucomannan Coatings

**Figure 2:**
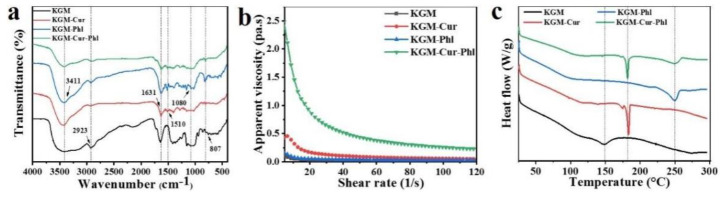
Characterization of konjac glucomannan coatings: (**a**) FTIR, (**b**) steady rheological behavior and (**c**) DSC.

FTIR is a characterization tool for qualitative and quantitative analysis of samples based on the principle of fourier transform of infrared light after interference. As shown in Figure 2a, the characteristic peaks of Cur were generally 1510 cm^−1^, 1631 cm^−1^, 2923 cm^−1^ and 3411 cm^−1^, which was assigned to the C=C stretch, C=O stretch, C-H stretch and O-H stretch, respectively [8]. The characteristic peaks of Phl generally appeared at 1080 cm^−1^ (C-O stretching), 1631 cm^−1^ (C=O stretching), 2923 cm^−1^ (C-H stretching) and 3411 cm^−1^ (O-H stretching). The characteristic peaks of KGM were 807 cm^−1^, 870 cm^−1^, 1732 cm^−1^, 2923 cm^−1^ and 3319 cm^−1^ [4]. The curve of KGM-Cur-Phl showed the characteristic peaks of Cur, Phl and KGM, which demonstrated the successful preparation of active coatings. Meanwhile, compared with the control group, the position and intensity of the hydroxyl absorption peak at 3411 cm^−1^ changed, indicating the hydrogen bond interaction was formed between the substances. The steady rheological behavior of coating solutions was investigated. As shown in Figure 2b, the apparent viscosity of coatings decreased as the shear rate increased, which demonstrated that coatings had pseudoplastic properties. The addition of Cur or Phl increased the apparent viscosity of KGM, and the apparent viscosity value of KGM-Cur-Phl was much greater than the sum of KGM-Cur and KGM-Phl. We also found that Phl and Cur were uniformly dispersed in KGM sol. Thus, the partial hydrogen bonding interaction between substances and the filling of KGM molecular chain by Cur and Phl could be responsible for this result [19]. The thermal stability of active coatings was further investigated by the DSC. As shown in Figure 2c, the different coatings exhibited different characteristic thermal absorption peaks: KGM was 150 °C, KGM-Cur was 180 °C and KGM-Phl was 250 °C. In the DSC curve of KGM-Cur-Phl, the heat absorption peaks from KGM-Cur and KGM-Phl can be obviously observed, which also indicated the successful preparation of the active coating. Meanwhile, compared with KGM, the thermal performance of KGM-Cur-Phl was improved, which was conducive to the continuous and stable activity of the coatings.

### 3.2. Antioxidant Activity Assay of Coatings

**Figure 3:**
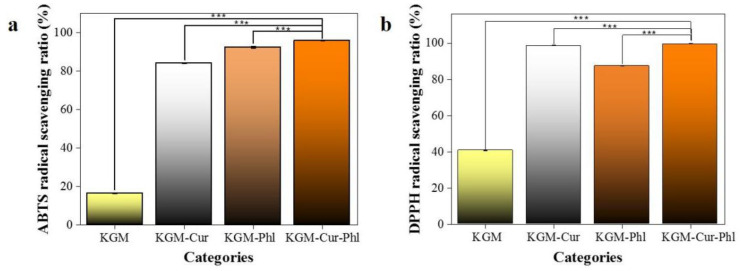
The antioxidant activities of konjac glucomannan coatings: (**a**) ABTS radical scavenging capacity, (**b**) DPPH radicals scavenging ratio. *** *p* < 0.001.

Cur is a polyphenol compound with antioxidant effects [20]. As a kind of flavonoid, Phl has antioxidant activity mainly in the hydroxyl group of aromatic hydrocarbons [11]. An ABTS radical scavenging experiment was carried out, and the corresponding results are shown in Figure 3a. Although KGM has a certain antioxidant capacity, the antioxidant capacity of pure KGM cannot meet practical application. The antioxidant capacity of the coatings was significantly improved by adding Cur or Phl into KGM. The ABTS radical scavenging ratio of KGM-Cur-Phl was 95.03%, which are conducive to the preservation of food [5]. In order to further verify the antioxidant effect of the experimental coatings, DPPH radical scavenging experiment was carried out. The experimental results of DPPH removal were basically consistent with ABTS, and the KGM-Cur-Phl value was as high as 99.67%. The above results fully show that the coating has excellent antioxidant activity.

### 3.3. Antibacterial Activity Assay of Coatings

**Figure 4:**
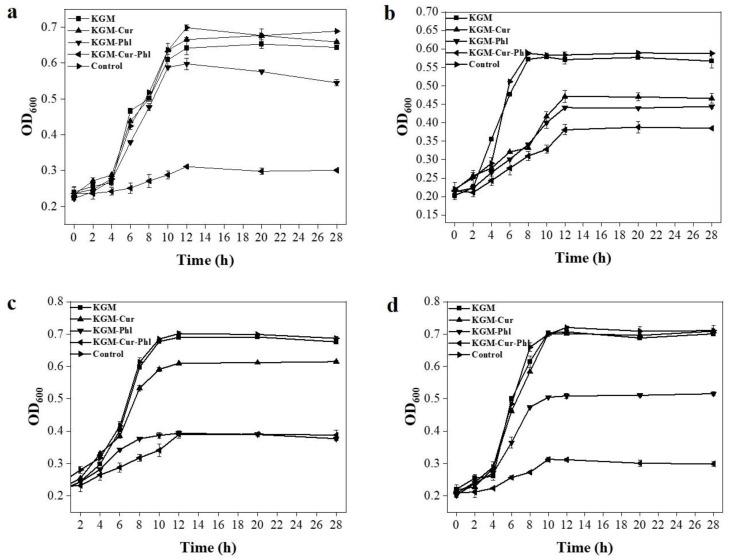
The antibacterial activity of konjac glucomannan coatings. The growth curve of (**a**) *L. monocytogenes*, (**b**) *S. typhimurium*, (**c**) *E. coli* and (**d**) *S. aureus*.

As a new type of green environmental protection, active coatings can not only protect the environment, but also protect food from bacteria, thereby maintaining its freshness. Previous studies have shown that Cur and Phl have antibacterial activities [21]. However, in practical application, the hydrophobicity of Cur and Phl reduce their antibacterial effect [22]. KGM has strong water solubility and can be used as an excellent carrier for hydrophobic active substances. The Gram-positive bacteria including L. monocytogenes and *S. aureus*, and Gram-negative bacteria including *E. coli* and *S. typhimurium*, were selected as the representative strains, and the corresponding bacterial growth curve was measured. The experimental results are shown in Figure 4. The curve of KGM and the control group showed no obvious downward trend, indicating that KGM had no antibacterial activity. Compared with KGM, the antibacterial activity of KGM-Cur and the KGM-Phl group was generally stronger. The antibacterial activity of KGM-Cur-Phl group was significantly higher than that of other groups. Referring to previous methods [23], the antibacterial efficiency of KGM-Cur-Phl was calculated at culturing with bacteria for 12 h. The results showed that the killing efficiency of KGM-Cur-Phl against *L. monocytogenes*, *S. typhimurium*, *E. coli* and *S. aureus* was 57.14%, 36.67%, 42.86% and 55.56%, respectively (Figure 4). These results showed that the coating was more sensitive to Gram-positive bacteria than Gram-negative bacteria. This may be due to the fact that Gram-negative bacteria has a stronger extracellular membrane than Gram-positive bacteria, thus reducing the killing effect of coatings, which is consistent with previous reports [24]. These phenomena show that Cur and Phl can effectively improve the antibacterial activity of pure KGM. The coatings with excellent antibacterial activity can prolong the shelf life of food by inhibiting the growth of microorganisms.

### 3.4. The Preservation Effect of Kumquats

**Figure 5:**
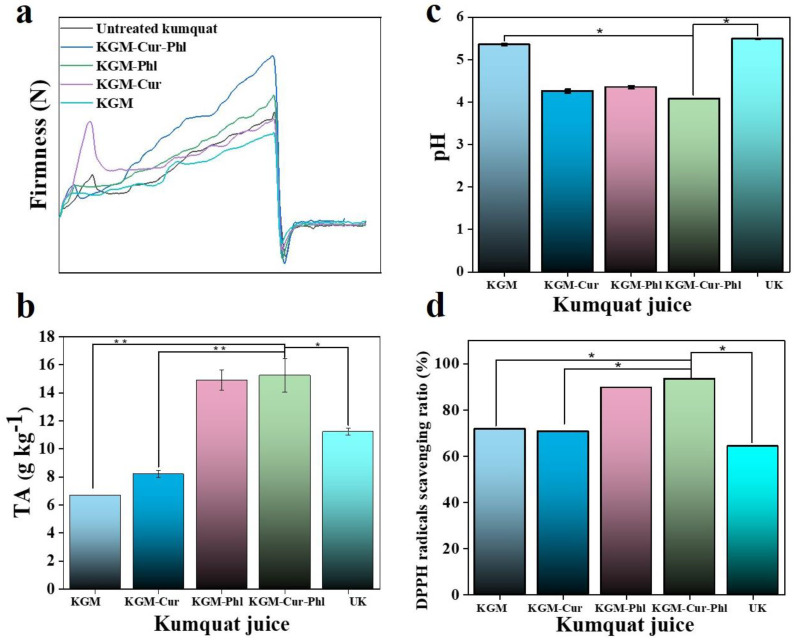
(**a**) Firmness of kumquats on day 12. (**b**) The titratable acid (TA) value of the corresponding kumquat juice. (**c**) The pH values of corresponding kumquat juice. (**d**) The DPPH radicals scavenging ratio of kumquats juice. UK means untreated kumquat. * *p* < 0.05, ** *p* < 0.01.

Since the KGM, Cur and Phl used in the coatings are edible natural active materials [25], we employed this active coating to slow down the deterioration of kumquat. There are great differences in the occurrence time and degree of kumquat corruption among kumquats treated with different active coatings. We measured the firmness of kumquat protected by different coatings. As shown in Figure 5a, the kumquats in KGM-Cur-Phl group showed the greatest firmness. It was reported that microbial or enzymatic reactions may cause loss of firmness in fresh produce during postharvest storage [26]. KGM-Cur-Phl can maintain the firmness of kumquats by effectively preventing bacterial infection, which also indicates that this coating has the best fresh-keeping effect. The titratable acid content is a significant component of fruit freshness, and it is also an important index to measure postharvest fruits without affecting the flavor qualities [18]. The primary reason for the decrease is that there is no external nutritional supplement for the fruits after picking, and the organic acid in the fruits are consumed as respiratory substrates. High titratable acid content in fruit indicates good freshness [27,28]. Therefore, titratable acid content and pH value of kumquat preserved for 12 days were measured to explore the preservation effect. As shown in Figure 5b, the titratable acid content of KGM-Cur-Phl treatment was the highest, which reflected the slow change in postharvest maturity and freshness [17]. The pH value measurement results showed that the pH value of KGM-Cur-Phl treatment was the lowest and the UK treatment was the largest among all groups (Figure 5c), which was the same for titratable acid measurements.

The DPPH radicals scavenging ratio of kumquat juice treated with different coatings was further measured to verify the freshness of kumquats. As shown in Figure 5d, compared with pure KGM treatment, the free radical scavenging ability of kumquats treated with KGM-Phl was improved. KGM-Cur-Phl treatment has the strongest free radical scavenging ability, which reflects that KGM-Cur-Phl treatment can slow down the oxidation process of kumquats, thus extending their shelf life. The results of firmness, titratable acid, pH and DPPH radicals scavenging ratio demonstrated that KGM-Cur-Phl could effectively prolong the shelf life of kumquats through its antioxidant and antibacterial activities.

## 4. Conclusions

In this study, a new active coating was prepared successfully by combining konjac glucomannan with curcumin and phloretin, which was confirmed by the fourier infrared spectroscopy, rheology and differential scanning calorimetry. The results of DPPH and ABTS radicals scavenging ratio demonstrated that it had excellent antioxidant activity. The growth curves of Gram-positive bacteria and Gram-negative bacteria showed that it had broad-spectrum antibacterial activity. Finally, kumquats were selected as the experimental objects to explore the role of active coatings in practical application. The results showed that this active coating could effectively prolong the shelf life of kumquat based on its excellent antioxidant and antibacterial activity. This work may be of great significance in the following aspects: a preparation method of polysaccharide-based active coating is provided; it broadens the application of low activity polysaccharides represented by konjac glucomannan in the field of food storage; it overcomes the application limitations of active substances such as curcumin and phloretin due to its low water solubility; and it provides a new attempt for the effective preservation of fruits and a reference for the evaluation of fruit freshness.

## Data Availability

The data presented in this study are available upon request from the corresponding author. The data can be offered to the proper persons with an objective goal for the use of the data.
